# The Levonorgestrel Intrauterine System Attenuates the Expression of Angiopoietin-1, Angiopoietin-2, and Vascular Endothelial Growth Factor in Adenomyosis

**DOI:** 10.3390/jcm14248629

**Published:** 2025-12-05

**Authors:** SiHyun Cho, Hyun Kyung Kim, Young Sik Choi, Joo Hyun Park

**Affiliations:** 1 Department of Obstetrics and Gynecology, Gangnam Severance Hospital, Yonsei University College of Medicine, Seoul 06273, Republic of Korea; sihyuncho@yuhs.ac; 2 Institute of Women’s Life Medical Science, Yonsei University College of Medicine, Seoul 03722, Republic of Korea; 3 Department of Laboratory Medicine, Yonsei University College of Medicine, Seoul 03722, Republic of Korea; kalmiaria@yuhs.ac; 4 Department of Obstetrics and Gynecology, Severance Hospital, Yonsei University College of Medicine, Seoul 03722, Republic of Korea; yschoi08@yuhs.ac; 5 Department of Obstetrics and Gynecology, Yongin Severance Hospital, Yonsei University College of Medicine, Yongin 16995, Republic of Korea

**Keywords:** adenomyosis, levonorgestrel intrauterine system, angiogenesis, VEGF, *ANGPT1*, *ANGPT2*

## Abstract

**Background/Objectives**: Adenomyosis is characterized by aberrant endometrial invasion and heavy menstrual bleeding, with angiogenesis being implicated as a key mechanism of this condition. We compared vascular endothelial growth factor (VEGF), angiopoietin-1 (ANGPT-1), and angiopoietin-2 (ANGPT-2) expression in eutopic and ectopic endometria from patients with adenomyosis and evaluated whether the levonorgestrel intrauterine system (LNG-IUS) modulates these angiogenic markers. **Methods**: In a case–control analysis, specimens from patients with adenomyosis without an LNG-IUS (*n* = 20), those with adenomyosis with prior LNG-IUS insertion (*n* = 18), and controls (*n* = 12) were analyzed. Immunohistochemistry with H-scores was used to assess protein expression in eutopic and ectopic tissues. *ANGPT1*, *ANGPT2*, and *VEGFA* mRNA in eutopic endometrial tissue were quantified by qRT-PCR. **Results**: In untreated adenomyosis patients, ectopic endometria showed higher protein expression than eutopic tissue for ANGPT-1, ANGPT-2, and VEGF (all *p* ≤ 0.05). The LNG-IUS was associated with significantly lower expression of all three markers in both eutopic and ectopic tissue (all *p* < 0.01), with eutopic levels approaching those of controls. qRT-PCR findings corroborated the decrease in *ANGPT1*, *ANGPT2*, and *VEGFA* transcript levels after LNG-IUS insertion (all *p* < 0.05). **Conclusions**: Adenomyosis is characterized by upregulated angiogenic signaling in both eutopic and ectopic endometria. The LNG-IUS attenuates ANGPT-1, ANGPT-2, and VEGF expression at both the protein and transcript levels, suggesting that modulation of angiogenic pathways may contribute to its therapeutic benefit in abnormal uterine bleeding associated with adenomyosis.

## 1. Introduction

Adenomyosis is characterized by aberrant infiltration of the endometrium into the adjacent myometrium, leading to dysmenorrhea, menorrhagia, and, in some patients, pregnancy-related complications. Its reported prevalence varies widely by ascertainment method—ranging between approximately 8% and 61% in hysterectomy-based series and about 20% and 34% in imaging-based clinical cohorts—whereas a United States population-based analysis estimated an increase in incidence of approximately 1% per year [1,2]. Adenomyosis and endometriosis are considered variants of the same disease, both involving the disruption of endometrial integrity, although the exact pathophysiology remains incompletely understood [3]. In adenomyosis, implants infiltrate deeply into the myometrium with irregular margins, and uterine-sparing surgical excision is rarely feasible. Consequently, conservative alternatives such as hormone therapy, uterine artery embolization, and the levonorgestrel intrauterine system (LNG-IUS) are important therapeutic options. The LNG-IUS, which initially releases approximately 20 µg/day (declining over time) within the uterine cavity, has been shown to reduce pain, bleeding, uterine volume, and anemia and is widely accepted as a nonsurgical alternative for adenomyosis management [4,5,6,7,8,9,10].

The endometrium is unique in undergoing rapid cycles of proliferation, degeneration, and regeneration, requiring dynamic vascular remodeling. Aberrant angiogenesis has been proposed as a key mechanism facilitating ectopic endometrial infiltration in adenomyosis, with recent systematic reviews highlighting its association with abnormal uterine bleeding and subfertility [11]. In endometriosis, ectopic lesions are often surrounded by richly vascularized tissue, and peritoneal fluid from affected women demonstrates increased angiogenic activity [11,12]. In addition, functional disturbances in adenomyosis, such as menorrhagia, have been associated with increased stromal vascular density [13]. Despite its clinical relevance, only limited information is available regarding the angiogenic factors that regulate endometrial vascular remodeling and how they are altered in adenomyosis [14,15,16]. Recent transcriptomic profiling supports this concept, showing that eutopic and ectopic endometrium in adenomyosis exhibits enrichment of angiogenesis-related pathways, including HIF-1α/VEGF signaling [17]. Furthermore, estrogen has been shown to upregulate VEGF—partly via the transcription factor Slug—thereby promoting vascular remodeling in adenomyosis [18].

Among these factors, vascular endothelial growth factor (VEGF) has been the most extensively investigated. VEGF is a glycoprotein that promotes endothelial cell growth, increases vascular permeability, and induces proteolytic enzymes critical for angiogenesis [19]. It is involved in the cyclic vascular changes observed in the endometrium, with expression increasing during the late secretory phase and menstruation and being upregulated under hypoxic or inflammatory stimuli. The overexpression of VEGF has been demonstrated in ectopic endometrial tissue in endometriosis [20].

The angiopoietins, particularly angiopoietin-1 (ANGPT-1) and angiopoietin-2 (ANGPT-2), are also central regulators of vascular remodeling. ANGPT-1, secreted by periendothelial support cells, binds to the TIE-2 receptor to stabilize vessels, maintain cell–cell interactions, and inhibit apoptosis [21,22,23]. In contrast, ANGPT-2 acts as a natural antagonist, destabilizing vessels by competitively binding to TIE-2, thereby loosening cell–cell and cell–matrix interactions and facilitating vascular remodeling in the presence of VEGF [24,25]. The balance between ANGPT-1 and ANGPT-2 expression determines whether endothelial cells quiescent or undergo proliferation or apoptosis remain [26].

Sex steroid hormones also modulate these angiogenic processes. Recent findings further support this interplay; pharmacologic inhibition of VEGF signaling substantially reduces lesion severity and angiogenic factor expression in an adenomyosis mouse model, highlighting the importance of hormone-responsive angiogenic pathways in disease progression [26].

Although the LNG-IUS is widely used for the conservative treatment of adenomyosis, the molecular mechanisms underlying its effects are poorly defined [26,27]. In particular, the impact of the LNG-IUS on endometrial angiogenic factors has not been systematically studied. Clarifying these mechanisms may provide insights into how the LNG-IUS alleviates abnormal uterine bleeding and pain.

The objective of this study was therefore to evaluate the expression of VEGF, ANGPT-1, and ANGPT-2 in eutopic and ectopic endometria from patients with adenomyosis—with and without LNG-IUS insertion—compared with normal controls. This analysis aimed to identify alterations in angiogenic factor expression associated with adenomyosis and to determine whether the LNG-IUS attenuates these changes.

## 2. Materials and Methods

### 2.1. Patient Selection

The study was reviewed and approved by the Institutional Review Board of Gangnam Severance Hospital, Yonsei University College of Medicine (IRB No. 3-2008-0215; approval date: 25 February 2009) under an exemption review. Because the study used surplus postoperative endometrial tissue that had been collected before the IRB approval date and subsequently archived in a fully de-identified manner through the Gangnam Severance Hospital Human-Derived Material Bank, the requirement for individual informed consent was formally waived. As the Biobank provides anonymized specimens without linked personal identifiers, the exact dates of tissue collection were not available. However, all specimens were fixed in 2009, and endometrial tissues exhibiting secretory phase morphology were primarily selected for analysis to minimize hormonal variation.

As this was an exploratory study, no formal sample size calculation was performed; instead, all eligible cases available in the Biobank and meeting the inclusion criteria were included. A total of 50 specimens were analyzed: 20 hysterectomy specimens with adenomyosis without other gynecologic pathology, 18 adenomyosis specimens from patients with prior levonorgestrel intrauterine system (LNG-IUS; Mirena^®^, Bayer HealthCare Pharmaceuticals Inc., Whippany, NJ, USA; 20 µg/day release) insertion, and 12 normal endometrial specimens as controls.

Only cases in which the LNG-IUS was removed intraoperatively or immediately before hysterectomy were included. Indications for LNG-IUS removal and subsequent hysterectomy included irregular bleeding, abdominal cramping, spontaneous expulsion, or foul-smelling vaginal discharge. Control endometrial tissues were obtained from patients with normal preoperative ultrasonographic findings and normal CA-125 levels.

The exclusion criteria included concomitant gynecologic disease, a history of smoking, prior hormone therapy, recent intrauterine device insertion other than the LNG-IUS, significant systemic illness, and a body mass index (BMI) > 25 kg/m^2^. Women with a current or past history of smoking were excluded, as cigarette smoking is known to modulate vascular and inflammatory pathways involved in VEGF/angiopoietin signaling, potentially acting as a confounding factor in immunohistochemical quantification [28]. Sections with clearly visible endomyometrial junctions were selected to allow the comparison of eutopic and ectopic endometria. Hysterectomy was performed via laparotomy or laparoscopy, and endometrial curettage was used for control samples.

### 2.2. Immunohistochemistry (IHC)

Formalin-fixed, paraffin-embedded tissue sections were deparaffinized in xylene, rehydrated through graded alcohols, and treated with 3% hydrogen peroxide for 10 min to block endogenous peroxidase activity. Antigen retrieval was performed by microwaving the samples in Tris-EDTA buffer (pH 9.0) for 15 min.

The primary antibodies were VEGF (polyclonal goat, 1:20; R&D Systems, Minneapolis, MN, USA), ANGPT-1 (polyclonal goat, 1:100; R&D Systems), and ANGPT-2 (polyclonal goat, 1:100; R&D Systems). Sections were incubated with primary antibodies for 90 min at room temperature, followed by the EnVision™ Detection Kit (Dako Cytomation, Carpinteria, CA, USA) for 30 min. Diaminobenzidine (DAB) was used as the chromogen, and Mayer’s hematoxylin was applied as the counterstain. Slides were mounted with synthetic resin and examined under light microscopy. Negative controls were processed in parallel with the omission of primary antibodies (and/or isotype control), and appropriate positive controls recommended by the manufacturer were included.

### 2.3. Assessment of Staining (H-Score)

Immunoreactivity was quantified using a modified H-score, calculated as:

H-score = Σ (*P_i_* × *i*),

where *i* represents the staining intensity (0 = none, 1 = weak, 2 = moderate, 3 = strong, 4 = very strong) and *P_i_* is the percentage of cells stained at each intensity (0–100%). The H-score ranges from 0 to 400; higher scores indicate stronger immunoreactivity. At least 30 endometrial glands per section were assessed.

Two experienced pathologists independently scored all slides in a blinded manner. Scores showing total agreement were used for analysis. Discrepancies were resolved through a joint consensus review with a third observer (a gynecologist) until total agreement was reached.

### 2.4. Quantitative Reverse-Transcription PCR (qRT-PCR)

Total RNA was extracted from frozen endometrial tissues using the RNeasy^®^ Plus Mini Kit (Qiagen, Hilden, Germany). One microgram of RNA was reverse-transcribed to cDNA using the iScript cDNA Synthesis Kit (Bio-Rad, Hercules, CA, USA). qRT-PCR was performed using Power SYBR^®^ Green Master Mix (Applied Biosystems, Foster City, CA, USA) on a StepOne Real-Time PCR System (Applied Biosystems). GAPDH served as the internal control, and the primer sequences for *ANGPT1*, *ANGPT2*, *VEGFA*, and *GAPDH* are listed in Table 1. Relative expression was calculated using the comparative ΔΔCt method, and all reactions were conducted in triplicate.

### 2.5. Statistical Analysis

Data were analyzed using SPSS software (version 21.0; IBM Corp., Armonk, NY, USA). Normality was assessed with the Shapiro–Wilk test, and continuous variables are presented as the mean ± SD. Within-patient comparisons of eutopic vs. ectopic endometria were analyzed using paired two-tailed *t*-tests (or Wilcoxon signed-rank tests when normality was violated). For comparisons across independent groups (control, adenomyosis without LNG-IUS, adenomyosis with LNG-IUS) within a given tissue, we used one-way ANOVA with Bonferroni post hoc tests (or Kruskal–Wallis tests with Dunn’s correction when normality was violated). A two-sided *p* < 0.05 was considered statistically significant. Figures were generated using GraphPad Prism (version 10.4.0; GraphPad Software, La Jolla, CA, USA).

## 3. Results

### 3.1. Patient Characteristics

No significant differences were observed in age, BMI, or parity among the adenomyosis, adenomyosis with LNG-IUS, and control groups. The mean duration of LNG-IUS insertion prior to removal was 5.43 ± 2.5 months. The baseline characteristics of the study population are summarized in Table 2. Indications for LNG-IUS removal and subsequent hysterectomy included prolonged unscheduled bleeding, abdominal cramping, spontaneous expulsion, or intolerable vaginal symptoms.

### 3.2. Immunohistochemical Expression of Angiogenic Factors

#### 3.2.1. ANGPT-1

**Figure 1:**
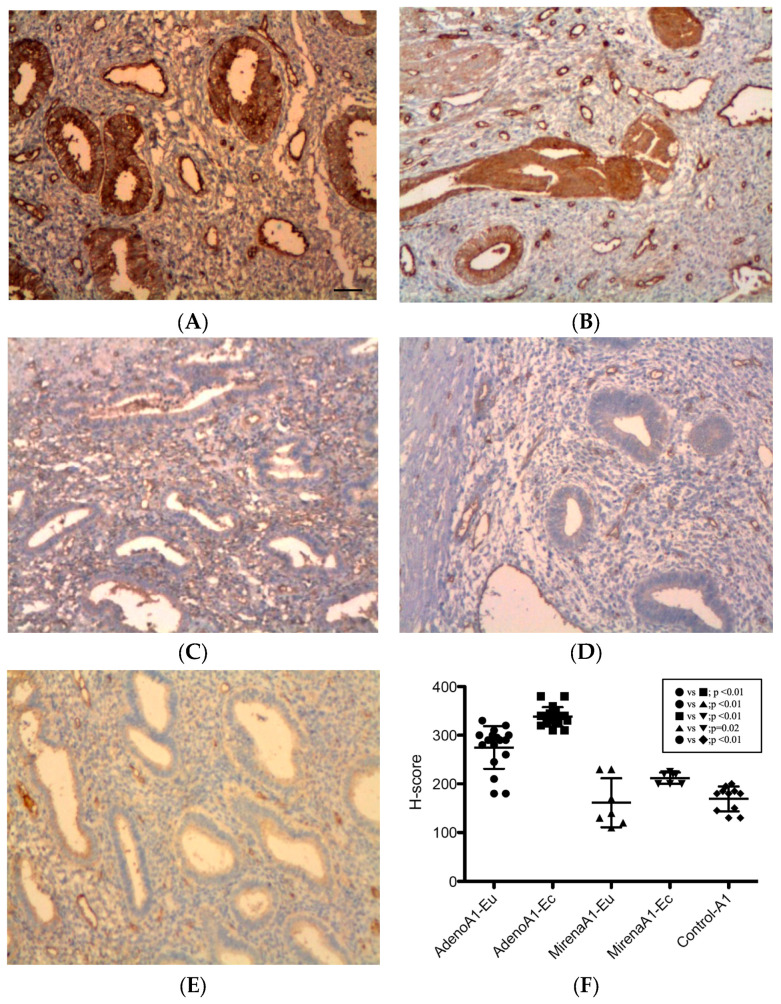
ANGPT-1 expression in the endometrium. Immunohistochemistry for ANGPT-1 showed predominant glandular epithelial cytoplasmic staining with additional periendothelial and stromal positivity. (**A**) Eutopic adenomyosis; (**B**) ectopic adenomyosis; (**C**) eutopic adenomyosis with LNG-IUS; (**D**) ectopic adenomyosis with LNG-IUS; (**E**) control endometrium; (**F**) semi-quantitative H-score analysis. In untreated adenomyosis, ectopic endometria exhibited higher ANGPT-1 levels than eutopic endometria (*p* < 0.01). LNG-IUS markedly reduced ANGPT-1 in both tissues (both *p* < 0.01), and LNG-IUS-treated eutopic levels were comparable with the controls (*p* = 0.67). Original magnification, ×100; scale bars, 100 µm. Data are the mean ± SD. Abbreviations: Adeno, adenomyosis; ANGPT-1, angiopoietin-1; Eu, eutopic; Ec, ectopic; LNG-IUS, levonorgestrel intrauterine system.

As shown in Figure 1, ANGPT-1 immunoreactivity was strongest in glandular epithelial cells, with additional focal staining in stromal and periendothelial tissues. In adenomyosis, ectopic endometria exhibited significantly higher expression than eutopic tissue (338.13 ± 19.94 vs. 272.86 ± 48.58, *p* < 0.01, Figure 1B vs. Figure 1A). In adenomyosis with administration of the LNG-IUS, a similar pattern was observed (212.14 ± 11.50 vs. 161.43 ± 50.47, *p* = 0.02, Figure 1D vs. Figure 1C). Compared with untreated adenomyosis, the LNG-IUS markedly reduced ANGPT-1 expression in both eutopic (161.43 ± 50.47 vs. 272.86 ± 48.58, *p* < 0.01, Figure 1C vs. Figure 1A) and ectopic tissues (212.14 ± 11.50 vs. 338.13 ± 19.94, *p* < 0.01, Figure 1D vs. Figure 1B). The eutopic endometria of adenomyosis patients demonstrated higher expression levels than the controls (272.86 ± 48.58 vs. 169.09 ± 25.48, *p* < 0.01, Figure 1A vs. Figure 1E), whereas the levels in LNG-IUS-treated eutopic samples were comparable to the controls (161.43 ± 50.47 vs. 169.09 ± 25.48, *p* = 0.67, Figure 1C vs. Figure 1E).

#### 3.2.2. ANGPT-2

**Figure 2:**
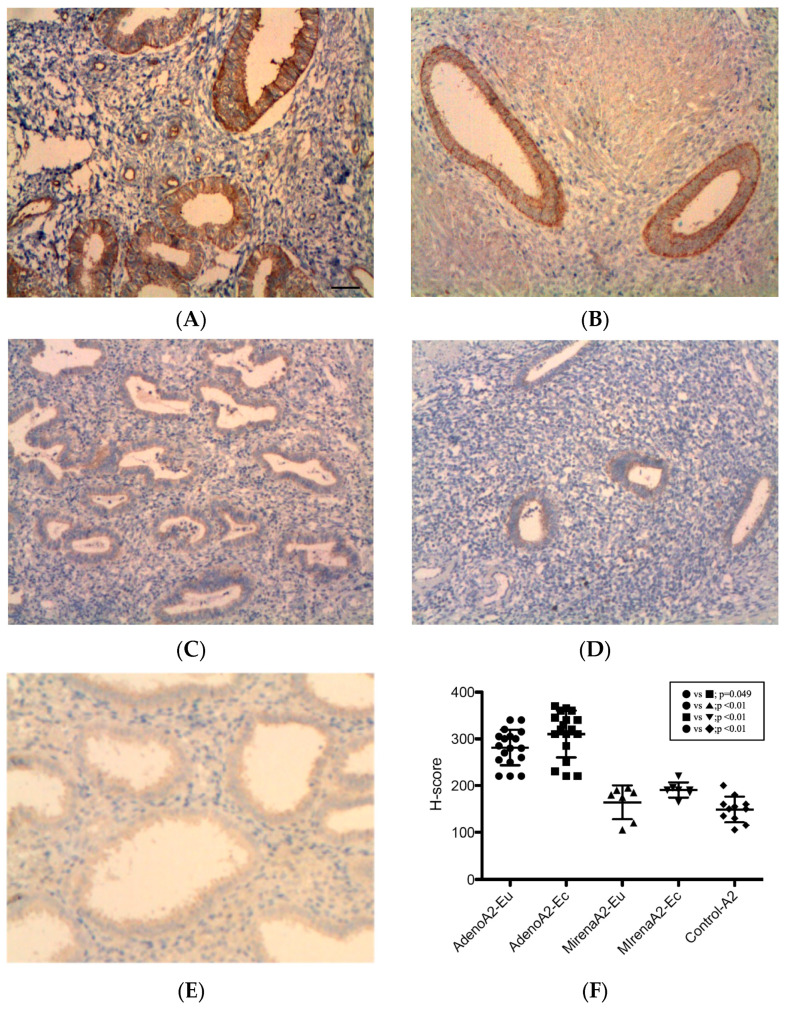
ANGPT-2 expression in the endometrium. ANGPT-2 immunoreactivity was strong in the glandular cytoplasm, with positivity in periendothelial/endothelial cells and focal stromal staining. (**A**) Eutopic adenomyosis; (**B**) ectopic adenomyosis; (**C**) eutopic adenomyosis with LNG-IUS; (**D**) ectopic adenomyosis with LNG-IUS; (**E**) control endometrium; (**F**) semi-quantitative H-score analysis. In adenomyosis patients, ectopic ANGPT-2 levels exceeded those in eutopic tissue (*p* = 0.049). With the LNG-IUS, the ectopic and eutopic levels did not differ (*p* = 0.10), and expression was reduced versus untreated adenomyosis in both compartments (both *p* < 0.01). Original magnification, ×100; scale bars, 100 µm. Data are mean ± SD. Abbreviations: ANGPT-2, angiopoietin-2; other abbreviations as in Figure 1.

All specimens were stained positive for ANGPT-2, with the strongest reactivity observed in the glandular epithelial cytoplasm and periendothelial and endothelial cells, while additional focal staining was observed in the stroma (Figure 2). In adenomyosis patients, the ectopic endometrium displayed significantly higher ANGPT-2 expression than eutopic tissue (310.32 ± 49.75 vs. 281.44 ± 38.26, *p* = 0.049, Figure 2B vs. Figure 2A,F). In adenomyosis patients with LNG-IUS insertion, no significant difference was noted between ectopic and eutopic tissue (190.71 ± 16.18 vs. 164.29 ± 36.22, *p* = 0.10, Figure 2D vs. Figure 2C,F). Compared with the adenomyosis without LNG-IUS group, expression was significantly reduced in both the eutopic (164.29 ± 36.22 vs. 281.44 ± 38.26, *p* < 0.01, Figure 2C vs. Figure 2A,F) and ectopic endometrium (190.71 ± 16.18 vs. 310.32 ± 49.75, *p* < 0.01, Figure 2D vs. Figure 2B,F). The eutopic endometrium of adenomyosis patients exhibited significantly higher ANGPT-2 expression compared with the controls (281.44 ± 38.26 vs. 149.09 ± 27.37, *p* < 0.01, Figure 2A vs. Figure 2E,F), whereas LNG-IUS-treated tissues showed levels comparable to the controls (164.29 ± 36.22 vs. 149.09 ± 27.37, *p* = 0.33, Figure 2C vs. Figure 2E,F).

#### 3.2.3. VEGF

**Figure 3:**
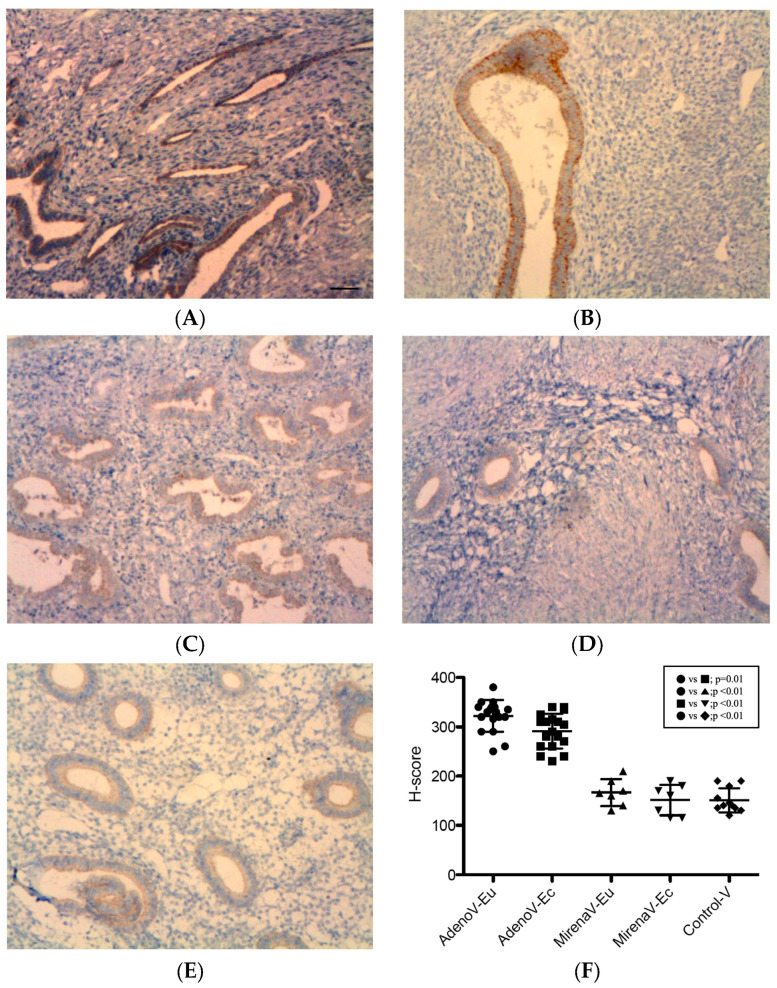
VEGF expression in the endometrium. VEGF localized mainly to the glandular epithelial cytoplasm and periendothelial/endothelial cells, with focal stromal staining. Reduced glandular density/size and staining intensity were observed after LNG-IUS. (**A**) Eutopic adenomyosis; (**B**) ectopic adenomyosis; (**C**) eutopic adenomyosis with LNG-IUS; (**D**) ectopic adenomyosis with LNG-IUS; (**E**) control endometrium; (**F**) semi-quantitative H-score analysis. In adenomyosis patients, ectopic VEGF levels exceeded those in eutopic tissue (322.01 ± 32.08 vs. 291.12 ± 35.38; *p* = 0.01). LNG-IUS reduced VEGF levels in both tissues versus untreated adenomyosis (both *p* < 0.01); LNG-IUS-treated eutopic levels were comparable with the controls (*p* = 0.23). Original magnification, ×100; scale bars, 100 µm. Data are mean ± SD. Abbreviations: VEGF, vascular endothelial growth factor; other abbreviations as in Figure 1.

VEGF immunoreactivity was detected in all specimens, with predominant localization to the glandular epithelial cytoplasm, as well as endothelial and periendothelial cells, and focal stromal staining (Figure 3). After LNG-IUS insertion, a reduction in glandular density, size, and staining intensity was noted (Figure 3C,D). In adenomyosis, VEGF expression was significantly higher in ectopic compared with eutopic tissue (322.01 ± 32.08 vs. 291.12 ± 35.38, *p* = 0.01, Figure 3B vs. Figure 3A,F). In the adenomyosis with LNG-IUS group, no significant differences were observed between ectopic and eutopic tissue (166.43 ± 27.47 vs. 151.43 ± 31.19, *p* = 0.36, Figure 3D vs. Figure 3C,F). Compared with the adenomyosis without LNG-IUS group, VEGF expression was significantly reduced in both eutopic (151.43 ± 31.19 vs. 291.12 ± 35.38, *p* < 0.01, Figure 3C vs. Figure 3A,F) and ectopic tissues (166.43 ± 27.47 vs. 322.01 ± 32.08, *p* < 0.01, Figure 3D vs. Figure 3B,F). The eutopic endometria of adenomyosis patients displayed significantly higher expression levels of VEGF compared with the controls (291.12 ± 35.38 vs. 150.91 ± 24.68, *p* < 0.01, Figure 3A vs. Figure 3E,F), whereas LNG-IUS-treated eutopic tissue showed no difference from the controls (166.43 ± 27.47 vs. 150.91 ± 24.68, *p* = 0.23, Figure 3C vs. Figure 3E,F).

### 3.3. mRNA Expression of ANGPT-1, ANGPT-2, and VEGFA

**Figure 4:**
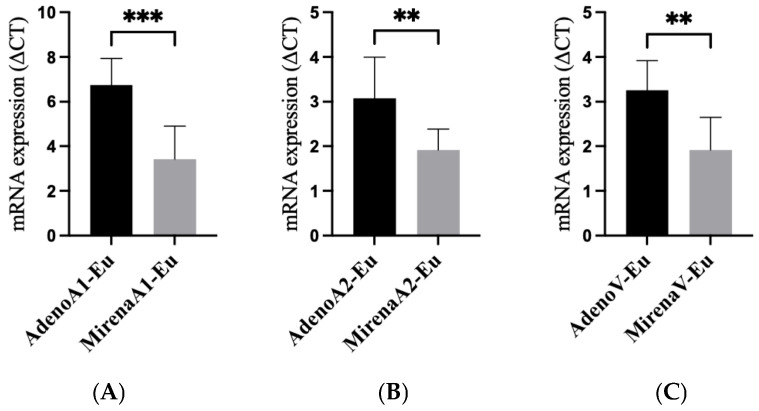
mRNA expression of angiogenic factors in the eutopic tissue of adenomyosis patients with and without LNG-IUS insertion. qRT-PCR of the eutopic endometrium showed LNG-IUS-associated suppression of angiogenic transcripts. (**A**) *ANGPT1* mRNA; (**B**) *ANGPT2* mRNA; (**C**) *VEGFA* mRNA (ΔΔCt). Bars represent the mean ± SD. *p* values: *ANGPT1*, 0.015; *ANGPT2*, 0.019; *VEGFA*, 0.028. Statistical comparisons were two-sided (parametric or non-parametric as appropriate). ** *p* < 0.01, *** *p* < 0.001. Abbreviations: qRT-PCR, quantitative reverse-transcription PCR; other abbreviations as in Figure 1.

qRT-PCR analysis demonstrated that LNG-IUS treatment significantly reduced the mRNA expression of all three angiogenic factors in the eutopic endometrium compared with untreated adenomyosis (*ANGPT1* mRNA: *p* = 0.015; *ANGPT2* mRNA: *p* = 0.019; *VEGFA* mRNA: *p* = 0.028, Figure 4A–C). The primer sequences are listed in Table 1.

Taken together, the immunohistochemical analyses demonstrated that the expression of *VEGFA*, *ANGPT1*, and *ANGPT2* was significantly elevated in both the eutopic and ectopic endometrium of adenomyosis patients compared with the controls, and that the LNG-IUS markedly reduced their expression in both tissues. Consistently, qRT-PCR in the eutopic endometrium confirmed the downregulation of *ANGPT1*, *ANGPT2*, and *VEGFA* after LNG-IUS insertion (Figure 4).

## 4. Discussion

Adenomyosis is a benign gynecologic disorder characterized by the infiltration of endometrial tissue into the myometrium, causing menorrhagia and dysmenorrhea. This infiltrative behavior resembles that of malignant tumors, raising the possibility that aberrant angiogenesis contributes to its pathogenesis [16,27]. A recent systematic review further highlighted the role of angiogenesis in adenomyosis, particularly its association with abnormal uterine bleeding and subfertility [11,29]. For the ectopic endometrium to establish and persist within the myometrium, angiogenesis, together with proteolytic activity, is considered essential. While angiogenesis has been extensively studied in endometriosis, relatively few reports have investigated its role in adenomyosis, and even less is known regarding the specific contributions of angiopoietins in this condition.

Among the angiogenic mediators, vascular endothelial growth factor (VEGF) has been the most widely investigated. VEGF is expressed in the human endometrium throughout the menstrual cycle, with peak expression during the late secretory and menstrual phases, likely as a response to hypoxia [20,30]. Estrogen is known to stimulate VEGF expression, although the role of progesterone remains less clearly defined [31]. In endometriosis, some studies have reported increased VEGF levels exclusively in ectopic implants, whereas others have observed elevated levels even in the eutopic endometrium [32,33]. Recent transcriptomic analyses in adenomyosis further support this concept by demonstrating enrichment of angiogenesis-related pathways—including HIF-1α/VEGF signaling—in eutopic and ectopic endometrium [17]. In addition, a 2024 mechanistic study reported that estrogen upregulates VEGF partly through the transcription factor Slug, promoting angiogenic activation in adenomyotic stromal cells [18]. Collectively, these data support the perception of angiogenesis as a central mechanism in adenomyosis pathophysiology [11,29] and suggest that molecular alterations in angiogenic signaling may precede, and potentially facilitate, the invasion of the endometrium into the myometrium.

The angiopoietins, particularly ANGPT-1 and ANGPT-2, also play central roles in vascular remodeling. ANGPT-1 typically stabilizes vessels through TIE-2 receptor binding, whereas ANGPT-2 antagonizes this effect and, in the presence of VEGF, promotes vessel destabilization and protease upregulation [34]. Although TIE-2 receptor expression itself remains stable during the menstrual cycle [35], our observation of increased ANGPT-1 and ANGPT-2 levels in adenomyosis patients indicates that altered ligand activity, rather than receptor availability, may contribute to aberrant angiogenesis. To our knowledge, this is the first study to describe the histologic distribution patterns of both angiopoietins in adenomyosis tissues and to examine their modulation in association with levonorgestrel exposure.

The levonorgestrel intrauterine system (LNG-IUS) is a widely used conservative treatment for adenomyosis and is well documented to reduce uterine bleeding and improve anemia with guideline support [36,37,38]. However, the molecular mechanisms underlying these effects remain poorly characterized. Our study demonstrates that LNG-IUS treatment is associated with lower protein expression of VEGF, ANGPT-1, and ANGPT-2 in adenomyosis, with levels approaching those observed in normal controls [39,40]. In parallel, qRT-PCR of eutopic endometria showed the downregulation of *ANGPT1*, *ANGPT2*, and *VEGFA* mRNA after LNG-IUS insertion. These findings suggest that decreased angiogenic signaling may be one mechanism associated with the clinical benefits observed with LNG-IUS use. These associations are biologically plausible, given that levonorgestrel locally activates progesterone receptors within the endometrium. This activity can antagonize estrogen-driven VEGF upregulation and reduce stromal inflammatory signaling. Notably, recent experimental data also support the potential therapeutic relevance of angiogenesis suppression in adenomyosis, as pharmacologic inhibition of VEGF signaling reduced lesion severity in vivo [26], aligning with the molecular trends observed in our study. While this study does not establish a direct link to clinical outcomes, the molecular evidence presented here provides a basis for future translational work to clarify how the LNG-IUS achieves symptomatic improvement in adenomyosis. qRT-PCR analysis was limited to eutopic endometrium because ectopic adenomyotic tissue was not available in sufficient quantity or purity for reliable RNA extraction, which may have restricted our ability to fully evaluate transcript-level changes across compartments.

Several limitations of this study should be acknowledged. First, the sample size was relatively small, reflecting the exploratory nature of this study. However, the observed downregulation of angiogenic factors is consistent with recent experimental findings demonstrating the therapeutic efficacy of suppressing angiogenesis in adenomyosis mouse models [26]. Second, immunohistochemistry provides spatial and semi-quantitative assessment but not absolute protein quantification; H-scores (range 0–400) are subject to observer variability, despite blinded dual scoring. Third, isolating pure ectopic endometrium embedded within myometrium is technically challenging, and contamination by surrounding tissue cannot be entirely excluded. Also, the use of curettage for controls versus hysterectomy for cases results in different tissue handling, which may potentially influence antigen preservation. Fourth, the mean LNG-IUS indwelling duration in our cohort was relatively short (5.43 ± 2.5 months Subgroup analyses based on duration were not feasible as patient information was blinded. Fifth, the cross-sectional case–control design limits causal inference. Although we selected samples based on morphological phase, we lacked precise menstrual dating or circulating hormone levels at tissue sampling, both of which influence angiogenic markers. Finally, clinical correlates (e.g., bleeding scores, pain, and hemoglobin) were not available, precluding direct molecular–symptom linkage.

Despite these limitations, the present study adds to evidence that angiogenesis is central to adenomyosis’ pathophysiology. Larger longitudinal studies with standardized cycle-phase sampling, quantitative proteomics, and integrated clinical outcomes will be important to confirm these findings and to determine whether angiogenesis-targeting strategies—successful in endometriosis models [41]—have a role in adenomyosis. Taken together, these findings should be interpreted as hypothesis-generating and warrant confirmation in larger longitudinal or mechanistic studies.

## 5. Conclusions

The protein expression of ANGPT-1, ANGPT-2, and VEGF was significantly elevated in both eutopic and ectopic endometria of patients with adenomyosis compared with normal controls. In adenomyosis patients using the LNG-IUS, these expression levels were lower and approached those of the control group, *ANGPT1*, *ANGPT2*, and *VEGFA* mRNA in eutopic endometria showed concordant downregulation. These findings support an association between LNG-IUS use and decreased angiogenic activity in adenomyosis and suggest that modulation of angiogenic factors may represent one pathway through which the LNG-IUS alleviates symptoms such as abnormal uterine bleeding. Future longitudinal and mechanistic studies are warranted to clarify these relationships. 

## Figures and Tables

**Table 1:** Primer sequences used for qRT-PCR.

Gene	Direction	Primer Sequence (5′-3′)	Accession No.	Amplicon (bp)
*GAPDH*	Forward	GTCTCCTCTGACTTCAACAGCG	NM_002046.7	131
	Reverse	ACCACCCTGTTGCTGTAGCCAA		
*ANGPT1*	Forward	CAACAGTGTCCTTCAGAAGCAGC	NM_001199859.3	150
	Reverse	CCAGCTTGATATACATCTGCACAG		
*ANGPT2*	Forward	ATTCAGCGACGTGAGGATGGCA	NM_001118887.2	139
	Reverse	GCACATAGCGTTGCTGATTAGTC		
*VEGFA*	Forward	TTGCCTTGCTGCTCTACCTCCA	NM_001025366.3	126
	Reverse	GATGGCACTAGCTGCGCTGATA		

Abbreviations: *VEGF*, vascular endothelial growth factor; *ANGPT*, angiopoietin; *GAPDH*, glyceraldehyde-3-phosphate dehydrogenase.

**Table 2:** Baseline characteristics of the study population.

Characteristic	Adenomyosis	Adenomyosis-LNG-IUS	Control Endometrium	*p*-Value
N	20	18	12	NA
Age (years)	45.33 ± 3.35	43.40 ± 4.62	41.73 ± 7.81	0.25
BMI (kg/m^2^)	23.11 ± 2.32	23.24 ± 2.81	21.99 ± 2.89	0.47
Parity	2.07 ± 1.10	1.60 ± 0.70	1.64 ± 0.81	0.36
Duration of LNG-IUS insertion (months)	NA	5.43 ± 2.5	NA	NA

Control endometria were obtained from patients who underwent diagnostic endometrial sampling and showed no pathological abnormalities. Values are mean ± SD unless otherwise indicated. *p*-values are from one-way ANOVA across the three groups. Abbreviations: BMI, body mass index; LNG-IUS, levonorgestrel intrauterine system; SD, standard deviation; NA, not applicable.

## Data Availability

The data supporting the findings of this study are included in the article. Additional information is available from the corresponding author upon reasonable request.

## Abbreviations

The following abbreviations are used in this manuscript:

ANGPT	Angiopoietin
ANGPT-1	Angiopoietin-1
ANGPT-2	Angiopoietin-2
BMI	Body mass index
CA-125	Cancer antigen 125
Ec	Ectopic endometrium
Eu	Eutopic endometrium
H-score	Histochemical immunoreactivity score
LNG-IUS	Levonorgestrel intrauterine system
VEGF	Vascular endothelial growth factor
TIE-2	Tyrosine kinase with immunoglobulin-like and EGF-like domains 2 receptor
